# The content of mutant EGFR DNA correlates with response to EGFR-TKIs in lung adenocarcinoma patients with common EGFR mutations

**DOI:** 10.1097/MD.0000000000003991

**Published:** 2016-07-01

**Authors:** Ming-Szu Hung, Jr-Hau Lung, Yu-Ching Lin, Yu-Hung Fang, Meng-Jer Hsieh, Ying-Huang Tsai

**Affiliations:** a Division of Thoracic Oncology, Department of Pulmonary and Critical Care Medicine, Chang Gung Memorial Hospital, Chiayi Branch, Puzi City; b Department of Medicine, College of Medicine, Chang Gung University, Taoyuan; c Department of Respiratory Care, Chang Gung University of Science and Technology, Chiayi Campus, Chiayi; d Department of Medical Research, Chang Gung Memorial Hospital, Chiayi Branch, Puzi City; e Division of Pulmonary Infection and Critical Care, Department of Pulmonary and Critical Care Medicine, Chang Gung Memorial Hospital, Chiayi Branch, Puzi City; f Department of Respiratory Care, College of Medicine, Chang Gung University, Taoyuan, Taiwan, ROC.

**Keywords:** EGFR, EGFR-TKI, lung cancer

## Abstract

This study aimed to elucidate the association of the content of mutant epidermal growth factor receptor (EGFR) deoxyribonucleic acid (DNA) with the treatment response to EGFR-tyrosine kinase inhibitor (TKI) and survival in patients with lung cancer.

This retrospective cohort study included 77 lung adenocarcinoma patients with common EGFR mutations from December 2012 to February 2015. The content of mutant EGFR DNA in lung cancer tissues was determined using an Amplification Refractory Mutation System. The association of the amount of mutant EGFR DNA with treatment response, the clinical variables, and the progression-free survival (PFS) after EGFR-TKI therapy were evaluated.

Using the amount of mutant EGR DNA above 4.77% as the cut-off value, the sensitivity to predict EGFR-TKI responder is 82.0% and the specificity is 75.0% (area under the curve [AUC]: 0.734, *P* = 0.003). The high content of mutant EGFR DNA is an independent factor associated with the response to EGFR-TKIs (odds ratio: 13.07, 95% confidence interval [CI]: 3.23–52.11, *P* = 0.0003). A significantly longer PFS was observed in the group with the high content of mutant EGFR DNA (26.3 months, 95% CI: 12.2–26.3) compared with the low content of mutant EGFR DNA groups (12.3 months, 95% CI: 5.7–14.8, *P* = 0.0155). A better predictive value of the content of mutant EGFR DNA was noted in patients with exon 19 deletions (AUC: 0.892, *P* < 0.0001) than exon 21 L858R mutations (AUC: 0.675, *P* = 0.0856).

Our results show that the content of mutant EGFR DNA is associated with the clinical response to EGFR-TKIs, especially in patients with exon 19 deletions mutation.

## 1 Introduction

The epidermal growth factor receptor (EGFR) pathway is an attractive target for lung cancer therapy, because EGFR signaling pathway plays an important role in the growth, proliferation, and survival of many solid tumors, including nonsmall cell lung cancer (NSCLC).^[1]^ A subgroup of patients with NSCLC having specific mutations in the tyrosine kinase domain of the EGFR gene, which correlates with favorable clinical responsiveness to EGFR tyrosine kinase inhibitor (EGFR-TKI) therapy, has been noted.^[2]^ All mutations appear to be limited to exons 18, 19, 20, and 21 of the EGFR gene^[3]^ and are most frequently found in patients with lung adenocarcinoma.^[4]^

EGFR mutations have been found in fewer than 10% of non-Asian patients with NSCLC,^[5]^ and in 30% of East Asians’ patients.^[6]^ Missense mutations in exon 21 (L858R) and in-frame deletions within exon 19 have been shown to be the most frequent EGFR-TKI sensitive mutations (80%) in NSCLC.^[7]^ Both exon 19 deletion and exon 21 missense mutations are common EGFR mutations and have been proved to be associated with a favorable response to first-line treatment with gefitinib^[8]^ as well as other EGFR-TKIs such as erlotinib^[9]^ and afatinib^[10]^ compared with standard chemotherapy in patients with NSCLC. In contrast, NSCLC tumors with wild-type EGFR receptors often do not respond to EGFR inhibitor therapy and actually are more responsive to traditional chemotherapy.^[8]^ As a result, identifying EGFR mutation status before initiation of EGFR-TKI therapy is advocated for patients with NSCLC.^[11]^

Intratumoral heterogeneity, which is due to cancer cells with different genetic alterations in a tumor tissue, could contribute to resistance to anticancer drugs.^[12]^ Intratumoral heterogeneity of EGFR mutations has been reported to be a potential source of treatment failure and drug resistance to EGFR-TKIs.^[12,13]^ Further studies have also shown that the mutant EGFR content is associated with the treatment response to EGFR-TKI.^[14]^ As a result, we propose that the content of mutant EGFR deoxyribonucleic acid (DNA) could be used in the prediction of the treatment response to EGFR-TKIs in patients with NSCLC.

In this study, we attempted to determine the content of mutant EGFR DNA in lung cancer cells and NSCLC tissues using the Therascreen EGFR RGQ PCR kit (Qiagen, Hilden, Germany). Then, we evaluated the association of the content of mutant EGFR DNA with the treatment response to EGFR-TKI and survival in advanced lung adenocarcinoma patients with common EGFR mutations.

## 2 Materials and methods

### 2.1 Patients and study design

This study is a retrospective cohort study. After being approved by the Institution Review Board of Chang Gung Memorial Hospital, we evaluated 77 patients with lung adenocarcinoma in Chang Gung Memorial Hospital, Chiayi Branch diagnosed from December 2012 to February 2015. All patients were treatment-naive stage IIIB or IV advanced stage patients with lung adenocarcinoma by American Joint Committee on Cancer 7th Edition staging criteria. EGFR mutation status in 18, 19, 20, and 21 exons of the EGFR gene were determined by Therascreen EGFR RGQ PCR kit (Qiagen). Patients with exon 19 deletions and exon 21 L858R mutations were included in our study and patients with exon 18 or exon 20 mutations were excluded from our study. The clinical variables of these patients were analyzed. Patients were treated with gefitinib (250 mg/d), erlotinib (150 mg/d), or afatinib (40 mg/d) until the progression of disease. The response of lesions was evaluated by chest computed tomography, brain magnetic resonance imaging, or bone scan according to Response Evaluation Criteria in Solid Tumors (RECIST) 1.1,^[15]^ 3 months after the initiation of treatment. EGFR-TKI responders were classified as complete response or partial response (PR) and nonresponders were classified as stable disease (SD) or progressive disease (PD), 3 months after the initiation of EGFR-TKI therapy. Progression-free survival (PFS) refers to the time from the first treatment to PD or death in patients. Overall survival (OS) refers to the time from the diagnosis to the cause of death or patients were censored at last follow-up.

### 2.2 Cell culture and DNA extraction

Two NSCLC cell lines H1650 (ATCC CRL-5883) and H1975 (ATCC CRL-5908), and human embryonic kidney 293 cell line (HEK293, ATCC CRL-1573), were purchased from American Type Culture Collection (Manassas, VA). The HEK293 cell line has wild-type EGFR and both H1650 and H1975 cell lines have EGFR mutations (heterozygous exon 19 delE746-A750 for H1650, heterozygous exon 21 L858R, and exon 20 T790M for H1975^[16]^). Cells were grown in Roswell Park Memorial Institute growth media supplemented with 10% fetal calf serum, 30 ng/mL EGF, 10 U/mL penicillin, and 10 μg/mL streptomycin at 37°C and 5% CO_2_.

DNA was extracted from formalin-fixed paraffin embedded tumors using the QIAamp DNA FFPE Tissue Kit and from cell lines using the QIAamp DNA Mini Kit (Qiagen).

### 2.3 Determination of the percentage of mutant EGFR DNA

After extraction of DNA for cell lines, the mutant EGFR DNA was mixed with wild-type EGFR DNA at different percentages (0.5%, 2.5%, 5%, 10%, 20%, and 50%) in a final concentration of 5 μg/μL. EGFR mutation analysis for the mixed DNA samples was performed using the Therascreen EGFR RGQ PCR kit (Qiagen) according to manufacturer's manual.^[17]^ Delta Ct (dCt) is calculated as the difference between the mutation assay Ct and control assay Ct from the same sample. The correlation of dCt and the logarithmic values of the percentage of mutant EGFR DNA were then evaluated.

### 2.4 Statistical analysis

The Pearson χ^2^ test was used to elucidate the differences of categorical variables among different groups. Receiver operating characteristic (ROC) curves and the Youden index were used to determine optimal cut-off values. Survival analysis was performed with a Kaplan–Meier analysis and log-rank test. Multivariate analysis was performed by logistic regression. A value of *P* < 0.05 was regarded as statistically significant. All statistical tests were analyzed using the computer software MedCalc version 15 (MedCalc Software, Ostend, Belgium).

## 3 Results

### 3.1 Patient characteristics

In our study, 77 lung adenocarcinoma patients with common EGFR mutations were enrolled (Table 1). The median age was 67.7 years (41–92 years). Most patients were female gender (n = 40, 52%), never-smoker (n = 68, 88.3%), and stage IV (n = 72, 93.5%) patients. Among those patients, 34 (44.2%) patients were with exon 19 deletions and 43 (55.8%) patients were with exon 21 L858R missense EGFR mutations. Gefitinib (n = 45, 58.4%), erlotinib (n = 27, 35%), or afatinib (n = 5, 6.6%) EGFR-TKIs were used for the first-line therapy in those patients. Three months after EGFR-TKI treatment, tumor response to treatment was evaluated and PR in 61 (79.2%), SD in 8 (10.4%), and PD in 8 (10.4%) patients were observed. The overall response rate to EGFR-TKIs was 79.2% and the disease control rate was 89.6%. The median PFS for all first-line EGFR-TKI patients was 14.8 months (95% confidence interval [CI]: 12.2–26.3 months) and median OS was not reached. Of the 77 samples, 21 (27.3%) were obtained from bronchoscopy biopsy, 31 (40.3%) from transthoracic biopsy, 14 (18.2%) from surgical biopsy, 7 (9.1%) from pleural biopsy, and 4 (5.2%) from pleural effusion cytology. Rebiopsy was performed in 11 patients after progression of the disease and exon 20 T790M mutation was observed in 4 (36.4%) of 11 patients.

**Table 1:**
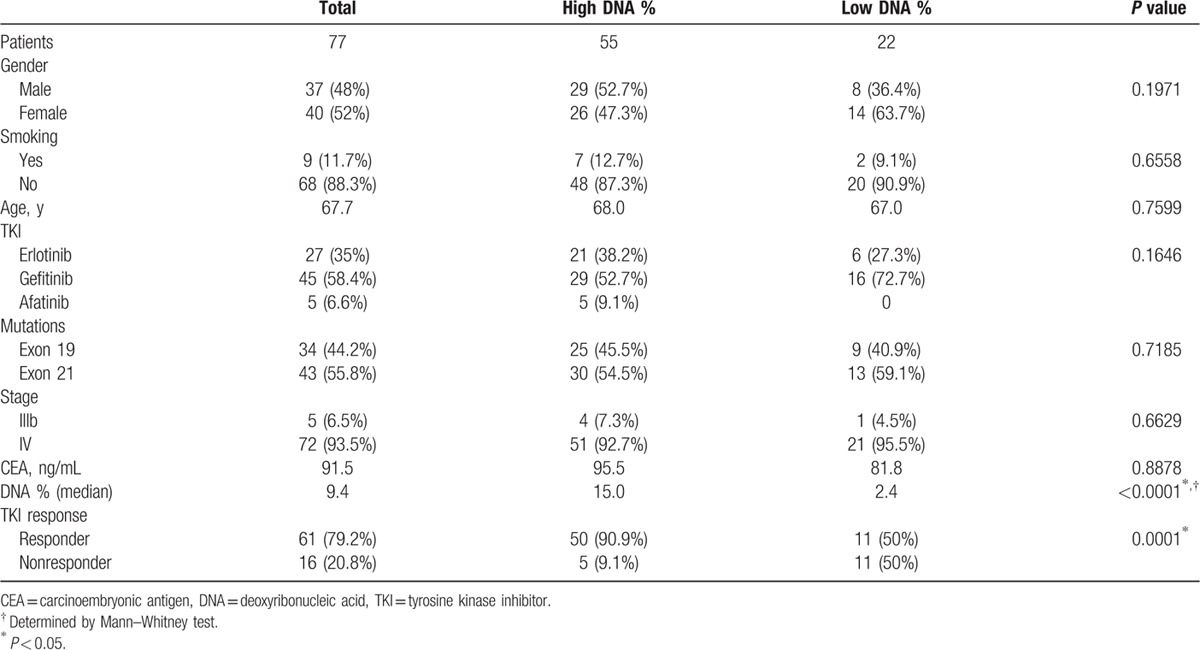
Patients’ characteristics and clinical variables.

### 3.2 The percentage of mutant EGFR DNA correlates with dCt

**Figure 1:**
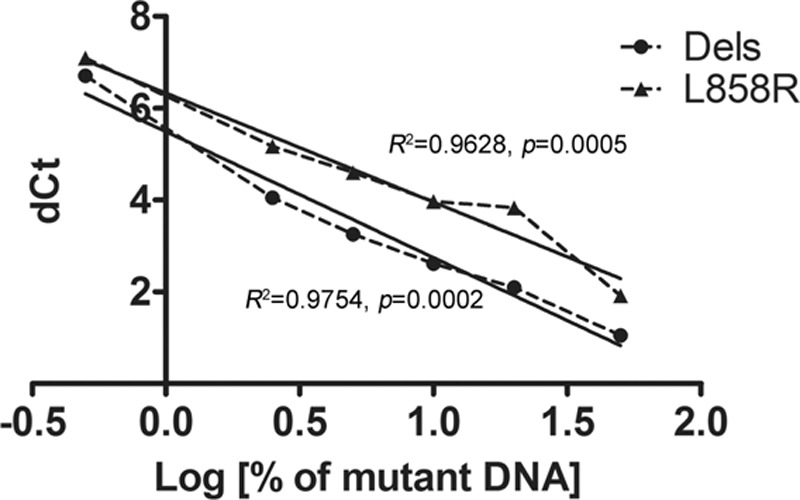
The association of the percentage of EGFR mutant DNA and delta Ct determined by the Therascreen EGFR RGQ PCR kit (Qiagen) in lung cancer cells. DNA = deoxyribonucleic acid, EGFR = epidermal growth factor.

To evaluate the association of dCt and the amount of mutant EGFR DNA, exon 19 deletions, and exon 21 L858R EGFR mutations were determined by the Therascreen EGFR RGQ PCR kit under different percentages of the mutant EGFR DNA. Liner regression was performed and a significant correlation was observed between the dCt and logarithmic values of the percentage of mutant EGFR DNA in both exon 19 deletions (R^2^ = 0.9754, *P* = 0.0002, dCt = −2.743 log DNA % + 5.491) and exon 21 L858R (R^2^ = 0.9628, *P* = 0.0005, dCt = −2.377 log DNA % + 6.332) mutations (Fig. 1). The content of mutant DNA in tumor samples was then calculated according to equations as previously described. The median content of the mutant EGFR DNA in all lung cancer tissues tested was 9.41% (95% CI: 7.38–15.44). Less than 0.5% of the mutant EGFR DNA was observed in 2 samples (0.12% and 0.27%), and more than 50% of the mutant EGFR DNA was observed in 3 samples (110.0%, 148.5%, and 2440%). All 3 patients with the percentage of the mutant EGFR DNA content more than 50% showed PR after EGFR-TKI therapy.

### 3.3 The percentage of mutant EGFR DNA correlates with clinical response to EGFR-TKI and survival

**Figure 2:**
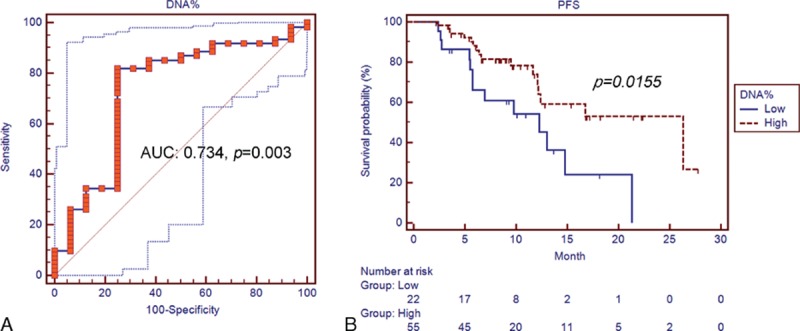
(A) ROC curve of using the amount of mutant EGFR DNA to predict EGFR-TKI responder in lung adenocarcinoma patients with exon 19 deletions and exon 21 L858R mutations. (B) PFS of the patients with high and low content of mutant EGFR DNA after EGFR-TKI therapy. DNA = deoxyribonucleic acid, EGFR-TKI = epidermal growth factor tyrosine kinase inhibitor, PFS = progression-free survival, ROC = receiver operating characteristic.

The sensitivity of the percentage of mutant EGFR DNA to predict clinical response to EGFR-TKIs was then evaluated. Using the percentage of mutant DNA above 4.77% as the cut-off value, the sensitivity to predict EGFR-TKI responder is 82% and the specificity is 75.0% (area under the curve [AUC]: 0.734, *P* = 0.003) (Fig. 2A). Logistic regression was performed to evaluate the relationship between the response to EGFR-TKIs and variables including age, sex, gender, EGFR mutation status, and mutant EGFR DNA content. The high content of mutant EGFR DNA is an independent factor associated with the response to EGFR-TKIs (odds ratio: 13.07, 95% CI: 3.23–52.11, *P* = 0.0003) (Table 2).

**Table 2:**
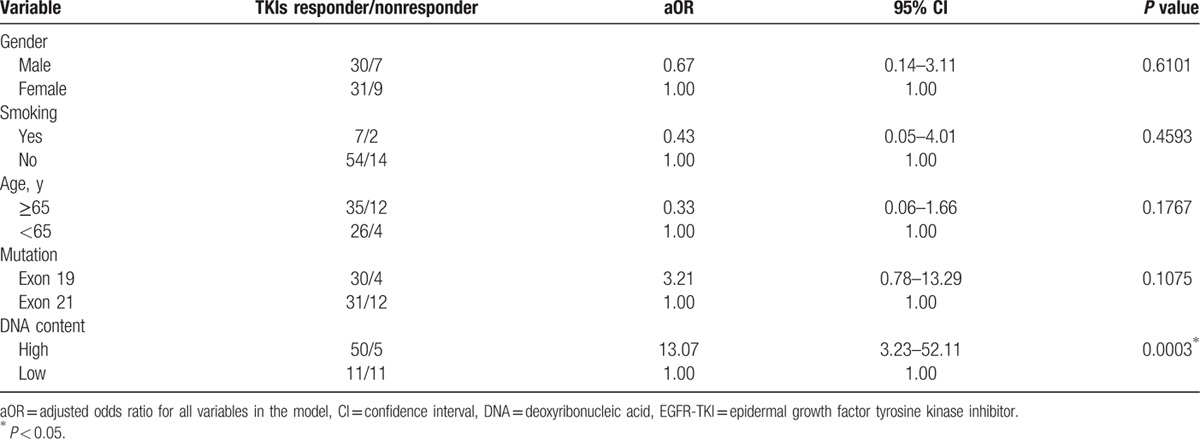
Multivariate logistic regression of the clinical variables and response to EGFR-TKIs.

The patients were then grouped into high and low content of the mutant EGFR DNA groups using 4.77% of mutant DNA as the cut-off value. A significantly higher proportion of EGFR-TKI responders was observed in high percentage of mutant DNA group compared with low percentage group (90.9% vs 50%, *P* = 0.0001) (Table 1). A significantly longer PFS was observed in the group with high content of mutant EGFR DNA (26.3 months, 95% CI: 12.2–26.3) compared with the low content of mutant EGFR DNA groups (12.3 months, 95% CI: 5.7–14.8, *P* = 0.0155) (Fig. 2B). There was no significant difference in gender, age, EGFR-TKI use, mutation status, and carcinoembryonic antigen levels between both groups.

**Figure 3:**
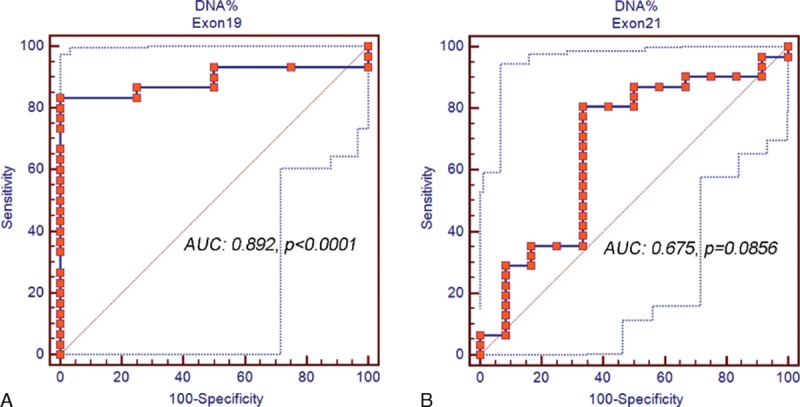
ROC curve of using the amount of mutant EGFR DNA to predict EGFR-TKI responder in lung adenocarcinoma patients with (A) exon 19 deletions and (B) exon 21 L858R mutations. DNA = deoxyribonucleic acid, EGFR-TKI = epidermal growth factor tyrosine kinase inhibitor, ROC = receiver operating characteristic.

**Figure 4:**
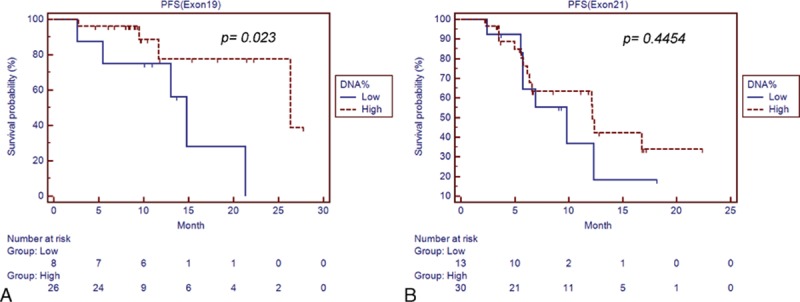
PFS of the patients with high and low content of mutant EGFR DNA after EGFR-TKI therapy in lung adenocarcinoma patients with (A) exon 19 deletions and (B) exon 21 L858R mutations. DNA = deoxyribonucleic acid, EGFR-TKI = epidermal growth factor tyrosine kinase inhibitor, PFS = progression-free survival.

The content of mutant EGFR DNA was further evaluated individually in exon 19 deletions and exon 21 L858R mutation patients. A better predictive value of the content of mutant EGFR DNA was noted in patients with exon 19 deletions (AUC: 0.892, *P* < 0.0001) (Fig. 3A) than exon 21 L858R mutation (AUC: 0.675, *P* = 0.0856) (Fig. 3B). Using the percentage of mutant DNA above 3.93% as the cut-off value, the sensitivity to predict EGFR-TKI responder is 83.33% and the specificity is 100% in patients with exon 19 deletion mutations. In those patients, a significantly longer PFS was observed in high percentage of mutant DNA groups (26.3 months, 95% CI: 26.3–26.3) compared with low percentage mutant DNA groups (14.8 months, 95% CI: 5.4–21.3, *P* = 0.023) (Fig. 4A). In patients with exon 21 L858R mutation, using the percentage of mutant DNA above 4.77% as the cut-off value, a longer PFS was observed in high percentage of mutant DNA groups (12.2 months, 95% CI: 6.4–16.8) compared with low percentage mutant DNA groups (9.8 months, 95% CI: 5.7–12.3, *P* = 0.4454) (Fig. 4B), although insignificant.

## 4 Discussion

In our study, we observed that the content of mutant EGFR DNA correlated with dCt detected by the Therascreen EGFR RGQ PCR kit (Qiagen) in lung cancer cells. In lung adenocarcinoma patients with common sensitizing EGFR mutations, the content of mutant DNA in lung cancer tissue samples correlated with the treatment response to EGFR-TKI. A higher percentage of mutant EGFR DNA was associated with a longer PFS after EGFR-TKI therapy. In addition, patients with exon 19 deletion mutations showed response to EGFR-TKI in lower contents of mutant EGFR DNA.

The Therascreen EGFR RGQ PCR kit assay is based on allele-specific amplification of mutant EGFR sequences using real-time polymerase chain reaction (PCR). It has been approved in the United States, Europe, and Asian countries with the purpose to detect of EGFR mutations with high sensitivity and specificity.^[17]^ PCR-based methods have been used in the determination of allele frequency in DNA samples.^[18]^ Thus, the Therascreen EGFR RGQ PCR kit assay was used in our study to determine the content of mutant EGFR DNA in lung cancer cells and tissues. From the literature review, we developed a method to quantify the content of mutant EGFR DNA using the Therascreen EGFR RGQ PCR kit assay for the first time.

Of EGFR-TKI nonresponders, 20% to 30% were noted in NSCLC patients with EGFR mutations receiving EGR-TKIs therapy.^[9,10,19]^ In EGFR-TKI nonresponders, inferior PFS and OS were observed compared with EGFR-TKI responders. A recent study reported that the median OS was 21 months (95% CI: 26.1–30.4) in responders compared with 8 months (95% CI: 8.7–15.8) in nonresponders.^[20]^ Since an inferior prognosis was noted in this group of patients, close monitoring for treatment response after the initiation of EGFR-TKI treatment is advocated with the purpose to identify EGFR-TKI nonresponders for early treatment adjustment. Our study results may help to further identify the EGFR-TKI nonresponders before the initiation of EGFR-TKI therapy.

Primary resistance to EGFR-TKI of lung cancer cells is related to EGFR-TKI nonresponders. Primary EGFR-TKI resistance has been reported to be related to v-Ki-ras2 Kirsten rat sarcoma viral oncogene homolog (KRAS) mutations,^[21]^ phosphoinositide-3-kinase catalytic alpha (PIK3CA) mutation,^[22]^ de novo proto-oncogene *MET* amplification,^[23]^ Bim deletion polymorphism,^[24]^ phosphatase and tensin homolog (PTEN) loss,^[25]^ and de novo T790M mutation of the EGFR gene.^[26]^ Our study may provide another evidence that low content of mutant EGFR DNA in lung cancer tissues may cause primary resistance to EGFR-TKI therapy in NSCLC patients with exon 19 deletions and exon 21 L858R EGFR mutations. However, a less correlation of the content of mutant EGFR DNA to the response of EGFR-TKIs was observed in patients with L858R mutation, which may imply that the resistance to EGFR-TKI may be regulated through different mechanisms in this group of patients. *MLH1* V384D polymorphism was reported to be associated with primary resistance to EGFR-TKI in lung adenocarcinoma patients with exon 21 L858R mutation.^[27]^ However, further study to elucidate the differences in mechanisms related to sensitivity to EGFR-TKI between L858R and exon 19 deletion mutations is still warranted.

A high content of mutant EGFR DNA is associated with increased response to EGFR-TKI therapy and PFS in our study. Increased copy number of the EGFR gene has been reported to be associated with increased response to EGFR-TKI therapy, PFS and OS.^[28,29]^ Copy number gain of the EGFR gene is associated with EGFR mutations in lung cancer cells^[30]^ and tissues.^[31]^ As the result, increased copy number of the EGFR gene may cause increased mutant EGFR DNA contents in EGFR mutation lung cancer tissues in our study. However, the association of the EGFR gene copy number and the content of mutant EGFR DNA in EGFR mutation lung cancer tissues still need further study.

In our study, exon 19 deletion mutations also showed favorable outcomes and responses to EGFR-TKI than L858R mutation in lower contents of mutant EGFR DNA. In previous studies, exon 19 deletion mutations have been reported to be associated with better outcomes than L858R mutations in patients with EGFR-TKI.^[32,33]^ Our study further showed that exon 19 deletions and L858R mutations are 2 distinct groups of patients, and different clinical treatment strategy may be considered in the future.

In summary, our results showed that the content of mutant EGFR DNA is associated with the clinical response to EGFR-TKIs in lung adenocarcinoma patients with common EGFR mutations, especially in patients with exon 19 deletion mutations. The content of mutant EGFR DNA could be used as an indicator to predict response to EGFR-TKI therapy. Our results also add another mechanism that low content of mutant EGFR DNA may cause the primary resistance of EGFR-TKIs in lung adenocarcinoma patients with common EGFR mutations.
